# Making Solar Hydrogen: A Review of the Challenges and Strategies of Synthesizing CuFeO_2_ Photocathodes for Photoelectrochemical Water Splitting

**DOI:** 10.3390/molecules30051152

**Published:** 2025-03-04

**Authors:** Mohamed El Idrissi, Bastian Mei, Mohammed Abd-Lefdil, Lahoucine Atourki

**Affiliations:** 1 Industrial Chemistry, Faculty of Chemistry & Biochemistry, Ruhr University Bochum, 44801 Bochum, Germany; mohamed.elidrissi@rub.de; 2 MANAPSE Lab, Faculty of Science, Mohammed V University, Rabat 1014, Morocco; m.abdlefdil@um5r.ac.ma (M.A.-L.); l.atourki@um5r.ac.ma (L.A.)

**Keywords:** water splitting, hydrogen production, delafossite, copper-based materials, semiconductors, nanomaterials, nanotechnology

## Abstract

Delafossite CuFeO_2_ has emerged as a promising earth-abundant p-type photocathode for solar fuel generation due to its stability in aqueous conditions and its favorable light absorption characteristics. However, practical photocurrent generation in CuFeO_2_ has consistently fallen short of its theoretical potential. This limitation is attributed primarily to suboptimal practical visible light absorption, resulting in diminished incident photon-to-current conversion efficiency (IPCE). Challenges related to charge separation and transport, originating from low acceptor density and inherent low conductivity, further contribute to the reported suboptimal performance of delafossite CuFeO_2_. Thus, the present review comprehensively documents the latest advancements in the field of CuFeO_2_ photocathode research, with a particular emphasis on strategies to overcome the challenges currently being faced and on the illustration of pathways that may lead to the enhancement of critical performance parameters such as photocurrents, photovoltage, and fill factor.

## 1. Introduction

Hydrogen, a carbon-free energy carrier, holds immense promise for various energy sectors, including industry and transportation, owing to its relatively high gravimetric energy density (143 MJ kg^−1^) [1,2,3,4]. The development of a global hydrogen economy is widely regarded as a critical pathway toward achieving energy sustainability and decarbonization, offering solutions to energy security, environmental degradation, and industrial decarbonization. However, technical and economic barriers such as high production costs, inefficient storage/delivery infrastructure, and reliance on fossil fuel-derived hydrogen must be urgently addressed. These challenges are compounded by escalating threats from climate change, including global warming, ocean acidification, and air pollution, which have intensified since the industrial revolution [5]. Hydrogen can be efficiently stored, transported, and utilized as fuel in eco-friendly fuel cells or combustion engines without generating harmful pollutants [6,7,8]. By 2050, an estimated 61,737 TWh_LHV_ of hydrogen will be required to fully defossilize the global energy-industry system, necessitating scalable and sustainable production methods [9]. Diverse methods for hydrogen production exist, including steam methane reforming [10], oil reforming [11], and coal and biomass gasification [12,13], as well as water splitting [14]. Today, approximately 90% of global hydrogen production relies on steam methane reforming and fossil fuel gasification. Nevertheless, the purity of hydrogen from these methods remains unsatisfactory, and its generation is generally accompanied by substantial CO_2_ emissions [15,16,17]. In contrast, electricity- or light-driven water splitting, an environmentally sustainable approach, has garnered significant attention as a sustainable means of hydrogen production.

Solar water splitting, which utilizes solar energy and abundant water resources, can be accomplished through the utilization of photovoltaic systems coupled with electrolysis (PV-E), photocatalytic (PC), and photoelectrochemical (PEC) water splitting [18]. While PV-E boasts high efficiency, its complexity results in higher production costs [19]. In contrast, PC and PEC systems, in which light-absorbing properties and the electrolyzers’ functionality are combined in a single device, potentially offer a more cost-effective approach. Of these, PEC systems enable separation of the oxygen evolution reaction (OER) and hydrogen evolution reaction (HER) at distinct electrodes, providing a commercially viable path to sustainable hydrogen production as costs continue to decline [20]. PC systems are expected to be the most cost-effective method for hydrogen production, but separating potentially explosive hydrogen–oxygen mixtures remains a major bottleneck. This concern arises because the OER and HER usually occur at the same material without spatial separation, and in the reactor, accumulation of both oxygen and hydrogen is likely to occur.

**Figure 1:**
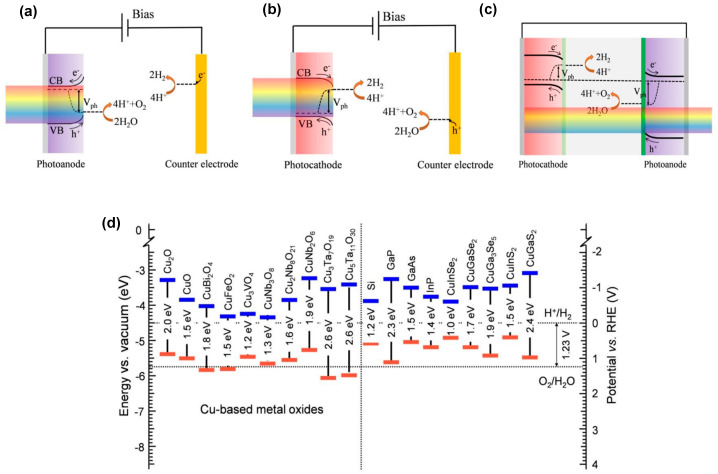
Principle of operation of PEC cells based on (**a**) bias-assisted n−type semiconductor, (**b**) bias-assisted p−type semiconductor, and (**c**) bias-free tandem structure consisting of p− and n−type photoelectrodes. (**d**) Energy band diagram of several Cu-based metal oxides together with a wide range of other semiconductors. The dotted lines represent the redox potentials of the relevant water splitting redox couples. Reprinted with permission from ref. [28], Copyright (2020), *Energy & Environmental Science*.

Various photoelectrochemical cell configurations for water splitting have been reported [21,22,23], with the simplest being composed of a single light-absorbing material. This setup includes a photoanode Figure 1a (or photocathode Figure 1b) made of an n-type (or p-type) semiconductor, along with a metal electrode working in the dark, serving as a cathode (or anode). Upon absorption of photons with sufficiently high energy, electron–hole pairs are generated, which are then separated within the semiconductor. The minority charge carriers initiate redox reactions at the semiconductor–liquid interface, while the majority carriers are directed toward the back contact and transported to the counter electrode, typically a metal, through an external circuit to support the other half-reaction. If a single absorber material (semiconductor) is used, it must absorb light with photon energies exceeding 1.23 eV to be able to split water without any additional applied bias. However, considering the well-known overpotentials of the hydrogen and oxygen evolution reaction, a bandgap of at least 2 eV is needed for efficient photoelectrochemical water splitting [22], limiting the use of the solar spectrum. To address this limitation, a more practical approach known as the tandem cell approach has been proposed (see Figure 1c). In tandem cell configurations, a pair of photo-absorbers, a photocathode, and a photoanode enable utilization of different parts of the solar spectrum and the generation of the required photovoltage for water splitting. Calculations have shown that a single absorber PEC system can achieve a maximum solar-to-hydrogen (STH) efficiency of 19.7%, while with tandem systems with two absorbers, STH values of 22.0% to 24.5% might be achievable [24]. To attain such high efficiencies, the photoelectrodes must meet several key requirements, including suitable band edge positions for water reduction and oxidation reactions and efficient charge transport. Moreover, the photoelectrode should facilitate hydrogen or oxygen evolution at low overpotentials, offer high chemical stability, and be prepared from low-cost reagents, i.e., nontoxic, and environmentally friendly earth-abundant materials, in a scalable synthesis method (possible materials are summarized in Figure 1d) [25,26,27].

According to estimates from the U.S. Department of Energy, achieving a solar-to-hydrogen (STH) efficiency of 10% is the fundamental requirement to reach commercial viability of photoelectrochemical (PEC) water splitting [29]. This benchmark efficiency equals to a photocurrent density of 8.2 mAcm^−2^ using standardized one-sun illumination (AM 1.5 G) [30], as revealed by the correlation of photocurrent density and STH efficiency expressed in Equation (1):

(1) $$STH\;efficiency=\frac{J\times 1.23\;V\times \eta_{Farady}}{P_{AM\;1.5G}},$$

where J represents the photocurrent density of the photoelectrode, $\eta_{Farady}$ signifies the Faradaic efficiency of water oxidation or proton reduction at the photoelectrode, and $P_{AM\;1.5G}$ denotes the power of solar irradiation (100 mWcm^−2^).

The overall efficiency of hydrogen production in PEC cells is predominantly determined by the characteristics of the photoelectrodes involved in the water-splitting processes. This efficiency (η) can be quantitatively influenced by three factors: (1) the solar light-harvesting efficiency of the photoelectrode ($\eta_{abs}$), (2) the charge transport efficiency within the photoelectrode ($\eta_{tran}$), and (3) the charge-transfer efficiency at the interface between the photoelectrode and the electrolyte ($\eta_{inter}$) [31]. In mathematical terms, this relationship can be expressed as follows:

(2) $$\eta =\eta_{abs}\times \eta_{tran}\times \eta_{inter},$$

The groundbreaking report on photoelectrochemical (PEC) water splitting, published in 1968, introduced the use of a thin layer of TiO_2_ with a band gap (E_g_) of approximately 3.2 eV as a photoanode in a PEC half-cell. Since then, considerable research efforts have been devoted to the development of photoelectrodes [32]. A range of n-type semiconductors, including n-Si (with an E_g_ of 1.1–1.3 eV) [33], TiO_2_ (with an E_g_ of 3.2–3.4 eV) [34], BiVO_4_ (with an E_g_ of 2.2–2.4 eV) [35], WO_3_ (with an E_g_ of 2.6–3 eV) [36], g-C_3_N_4_ (with an E_g_ of 2.5–2.8 eV) [37], CdS (with an E_g_ of 2.2–2.4 eV) [38], SrTiO_3_ (with an E_g_ of 3.2–3.4 eV) [39], and Fe_2_O_3_ (with an E_g_ of 2–2.2 eV) [40], have been extensively investigated for their application as photoanodes in PEC cells. Among these materials, TiO_2_ stands out for its stability, while BiVO_4_ excels in light absorption in the visible region [41]. It is worth noting that relatively little attention has been paid to the study of photocathodes, despite the extensive research into photoanodes. Efficient photocathodes for PEC water reduction include, among others, semiconductors like Si [42,43], InP [44], CdTe [45], CuIn_x_Ga_1−x_Se_2_ [46,47], CuInS_2_ [48], CuGa_3_Se_5_ [49], and CuGaSe_2_ [50,51]. However, the photovoltages generated by these photocathodes fall short of the thermodynamic potential of 1.23 V. The quest for high-photovoltage photocathodes is critical, particularly for constructing highly efficient two-electrode tandem devices alongside high-performance photoanodes, such as BiVO_4_ or n-Si, to enable bias-free water splitting. In addition, environmental and resource concerns, including the scarcity of indium and gallium and the toxicity of cadmium in CdTe, have driven the search for metal oxide-based photocathodes composed of earth-abundant and non-toxic elements that can be produced by cost-effective and scalable processes [52].

Cu-based binary and ternary metal oxides are promising absorber materials for PEC water reduction when compared to other semiconductor materials like group IV, III-V, and I-III-VI semiconductors. Cu-based oxides exhibit a wide range of bandgap values, ranging from 1.2 to over 3 eV, offering diverse options as efficient light absorbers for solar energy conversion [53]. The majority of Cu-based metal oxides feature conduction band edges situated above the hydrogen evolution potential, rendering the water reduction reaction thermodynamically feasible. Among these, binary oxides such as Cu_2_O and CuO have band gaps (2.0 and 1.2–1.5 eV) that are suitable for efficient solar light harvesting. However, these photocathodes suffer from photo-corrosion, mainly due to the unfavorable position of the redox couple for Cu_2_O and CuO reduction situated within the band gap of these semiconductors [54]. A strategic protection approach is therefore essential, with particular focus on interface engineering, band alignment, and carrier transport, all of which are of critical importance in enhancing the PEC performance of binary Cu-based heterojunction photocathodes [55,56].

**Figure 2:**
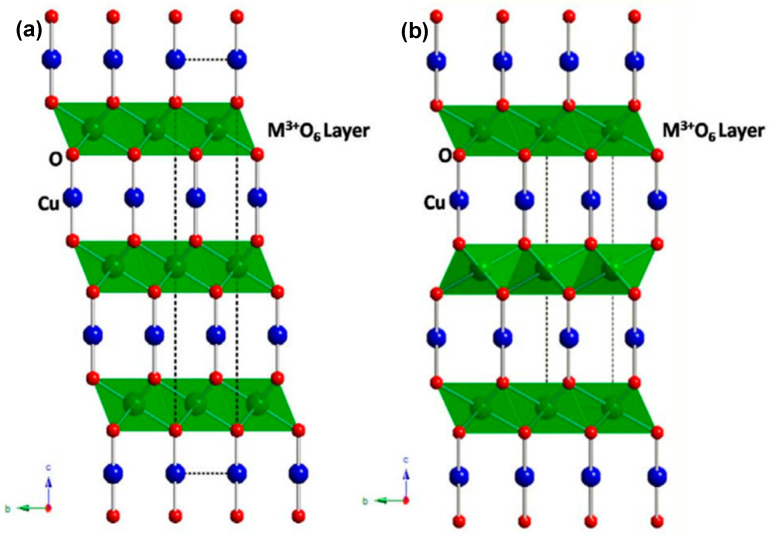
Two main polymorphic modifications of the delafossite structure that crystallize in the trigonal R$\bar{3}$m (**a**) and hexagonal P6_3_/mmc (**b**) space groups.

Copper-based ternary oxides offer a broader spectrum for fine-tuning band structure and optoelectronic properties in comparison with their binary counterparts. Among the ternary oxides, CuBi_2_O_4_ has been shown to be a promising photocathode material capable of harnessing visible light and generating substantial photovoltages [57]. With its band gap of approximately 1.8 eV and a reported photocurrent onset potentials of about 1 V vs. the reversible hydrogen electrode (RHE), CuBi_2_O_4_ is a material of interest for use in two-electrode tandem photoelectrochemical (PEC) devices [58]. The valence and conduction band edges of CuBi_2_O_4_ are composed of Bi 6s and Cu 3d orbitals, respectively [59]. However, in a manner analogous to Cu_2_O self-reduction, CuBi_2_O_4_ photocathodes are susceptible to photocorrosion, presumably as a consequence of the trapping of photoelectrons in the Cu 3d band [60]. Cu-containing delafossites bearing the general formula CuMO_2_, where M can be Fe, Rh, Cr, Al, Ga, In, or Sc, offer enhanced stability. These materials crystallize in either the rhombohedral (3R) or hexagonal (2H) polytypes, with space groups of R$\bar{3}$m (Figure 2a), and P6_3_/mmc (Figure 2b), respectively [61]. In particular, the valence and conduction band edges in Cu-containing delafossites are predominantly composed of Cu 3d and M d or s orbitals. This configuration limits the reduction of Cu(I) by Cu 3d→M d/s electron photoexcitation [62]. Furthermore, the light absorption characteristics of Cu-containing delafossites depend on the cation M and range from ~1.55 eV to band gaps exceeding 3 eV [62].

Among these Cu-containing delafossites, CuFeO_2_, with a small band gap of 1.55 eV, an absorption coefficient of up to α ~ 10^7^ m⁻^1^, a flat-band potential around 1 V vs. RHE, and its high stability in neutral and basic aqueous environments over extended periods [62], stands out as a promising photocathode material. Its unique combination of earth abundance (Cu and Fe are 50–100× more abundant than In/Ga) [63], non-toxicity, and scalable synthesis routes compared to conventional photocathodes like CdTe or InP makes it a strong candidate for sustainable hydrogen production systems. As summarized in Table 1, CuFeO_2_ offers notable advantages over state-of-the-art photocathodes in terms of performance, stability, and environmental impact, reinforcing its potential for large-scale hydrogen production. Beyond energy applications, CuFeO_2_′s stability and electronic properties have also enabled its use in catalytic degradation of pollutants, magnetic devices, and biomedical sensors [64,65,66]. However, despite these promising characteristics that theoretically enable photocurrents of up to 15 mA cm^−2^, the realized hydrogen evolution efficiencies of CuFeO_2_ have remain suboptimal [67]. Moreover, measurements utilizing oxygen as an electron scavenger called into question the assumption that CuFeO_2_ is an efficient PEC water-splitting material [68]. The objective of comprehending and enhancing this unsatisfactory photocurrent density has become a primary focus in CuFeO_2_ photocathode research. One of the main obstacles to the performance of CuFeO_2_ photocathodes has been identified as the pinning of the Fermi level at the solid–liquid interface [67,69]. Initially attributed to metal hydroxyl groups on the surface, these states, situated approximately 0.6 eV above the valence band, were later linked to the Fe^3+^/Fe^2+^ polaron. Recent studies of CuFeO_2_ photocathodes have highlighted additional challenges, including limited photogenerated electron diffusion lengths and polaron-mediated charge-carrier recombination [70].

This review focuses on CuFeO_2_ photocathodes, with a view to elucidating recent endeavors to enhance the material’s performance as a high-efficiency photocathode. We outline the merits and constraints of CuFeO_2_ as a p-type material, and strategies aimed at addressing challenges associated with the limited PEC performance are categorized. Key techniques that have been successfully applied to the development of CuFeO_2_-based solar cells and photocathodes will be discussed, thereby highlighting remaining bottlenecks. Additionally, we examine efforts to overcome these limitations and advance CuFeO_2_ toward practical implementation in photoelectrochemical solar fuel production.

## 2. Drawbacks of Bare CuFeO_2_ Photocathodes

Delafossite-phase CuFeO_2_ has garnered attention as a promising candidate for use as a photocathode, attributed to its intrinsic stability, high absorption coefficient, and a flat band potential of approximately 1 V_RHE_ [71,76,77]. Notably, the hybridization of Cu 3d with O 2p contributes to the valence band (VB), while the conduction band (CB) is composed of Cu 3d and Fe 3d states, leading to a narrow bandgap of around 1.5 eV [78]. The CB edge is situated just above the redox potential of the H^+^/H_2_ couple, resulting in favorable electron transfer conditions for hydrogen evolution [71]. Additionally, CuFeO_2_ exhibits a multi-band absorption spectrum, covering both ultraviolet (UV) and infrared (IR) regions. Despite the theoretically anticipated photocurrents of up to 15 mA cm^−2^ [77], the actual measured hydrogen evolution efficiencies for bare CuFeO_2_ have fallen considerably short of these expectations. Table 2 provides an overview of the reported performance of CuFeO_2_ photocathodes for solar hydrogen production, illustrating the discrepancy between theoretical predictions and experimental results.

CuFeO_2_-based photocathodes exhibit limited visible light harvesting, often resulting in low incident photon to current conversion efficiencies (IPCE) of >5% in the 600–800 nm wavelength range [3]. The relatively weak absorption and poor charge carrier transport near the absorption edge of this p-type oxide contributes to the poor IPCE [77]. In addition, it has been shown experimentally that the performance of CuFeO_2_ photocathodes is limited by poor charge separation and slow transport due to low acceptor density, resulting in a low conductivity [80,88].

Fermi-level pinning in iron-containing oxides like hematite (α-Fe_2_O_3_) and BiFeO_3_, linked to redox transition of Fe^3+^ to Fe^2+^, has been reported [89,90]. Similar Fermi-level pinning has been observed in CuFeO_2_ and was attributed to the presence of surface or bulk defect states [67,69]. Distinguishing between bulk and surface defects is crucial as their elimination requires different treatment strategies. While a high density of intrinsic bulk defects can affect a material’s suitability as a photoelectrode, surface defects are more suitable for post-processing strategies and surface modifications, such as the application of overlayers [91] or etching and regrowth strategies [92]. Thus, ongoing efforts aim at discerning whether CuFeO_2_′s performance stems from bulk or surface defects.

**Figure 3:**
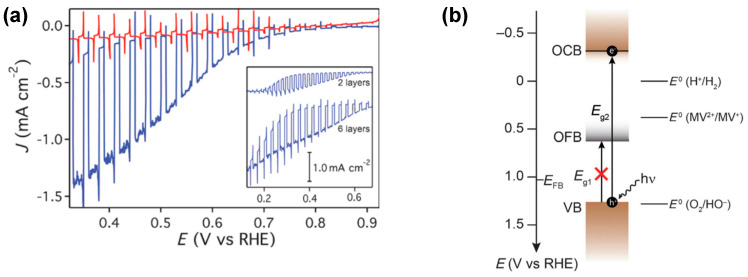
(**a**) Linear sweep voltammogram of a six−layer CuFeO_2_ electrode under intermittent one sun illumination in 1 M NaOH argon-purged electrolyte (red) and oxygen-saturated electrolyte (blue). Inset: wider potential range scans for two- and six-layer electrodes in oxygen-saturated electrolyte. (**b**) Band energy diagram of CuFeO_2_ under flat-band conditions. Reprinted with permission from ref. [71], Copyright (2015), *ChemSusChem*.

Prévot et al. [71] conducted an intriguing investigation into the performance of a thin film delafossite photocathode using front-side illumination. Their findings unveiled the presence of a smaller electronic band gap, corresponding to an optically forbidden excitation. Water splitting resulted in steady-state photocurrents of approximately 25 μA cm^−2^ at +0.4 V vs. RHE under chopped light illumination in an argon-purged electrolyte. However, in the presence of a sacrificial electron acceptor, such as oxygen, a dramatic increase in steady-state photocurrents to 1.05 mA cm^−2^ was observed (Figure 3a). Electrochemical impedance spectroscopy (EIS) revealed that the inversion layer of the p-type electrode was only 0.45 V cathodic to the flat band potential. It was suggested that there may be a smaller electronic band gap associated with an optically forbidden excitation. In their attempt to provide a rational explanation for their observations, Prévot et al. [71] highlighted the lower photocurrents for hydrogen evolution relative to O_2_ reduction alongside a less-than-unity Faradaic efficiency and suggested that oxygen reduction occurs from mid-gap electronic states. Crucially, the presence of mid-gap electronic states, detected by electrochemical impedance spectroscopy (EIS) (see Figure 3b), cast doubt on the ability of photogenerated carriers to effectively participate in water reduction under illumination. The rate of photogenerated electrons decaying into these low energy states must not exceed their injection into the electrolyte; otherwise, the potential of this material for water reduction would be impeded.

**Figure 4:**
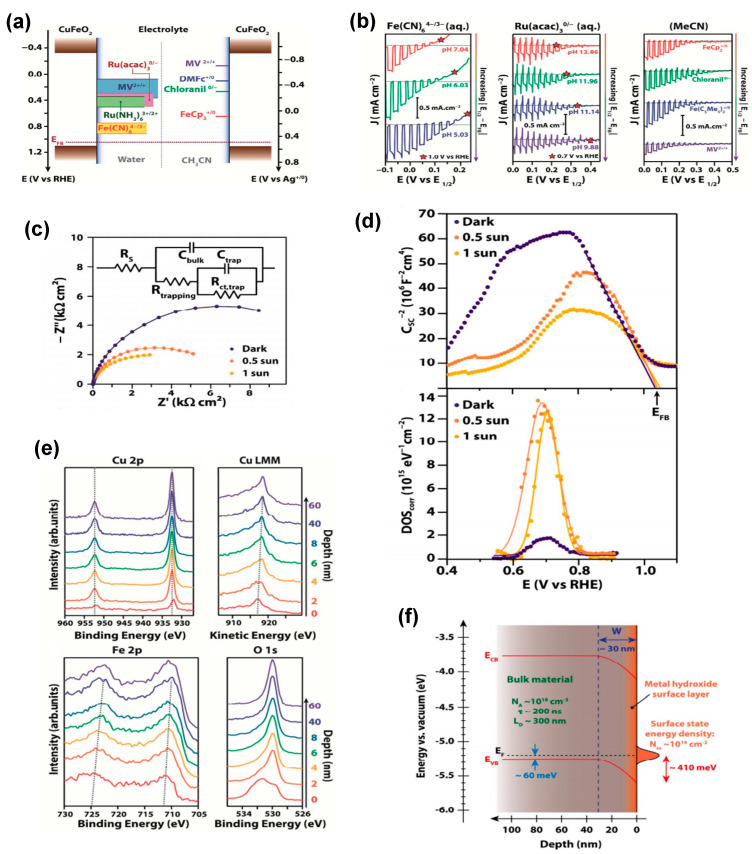
(**a**) Schematic representation of the potential regions probed by the different redox couples shown next to the band position of CuFeO_2_. (**b**) LSV curves of CuFeO_2_. (**c**) Typical Nyquist plots representing EIS data for a CuFeO_2_ electrode, along with the equivalent circuit used to fit them. (**d**) Mott−Schottky plots of CuFeO_2_ electrodes and the corresponding density of surface states (DOS) extracted from C_trap_. (**e**) XPS spectra as a function of depth of Cu 2p, Cu LMM, Fe 2p, and O 1s. (**f**) Proposed energy band diagram for an isolated CuFeO_2_ electrode in the dark. Reprinted with permission from ref. [67], Copyright (2017), *Chemistry of Materials*.

Further characterization of the semiconductor-liquid junction (SCLJ) of CuFeO_2_ using different redox couples in aqueous electrolytes of different pH and in non-aqueous electrolytes (see Figure 4a,b) confirmed a Nernstian behavior of the electronic bands of CuFeO_2_ over a narrow potential range of 0.77–0.90 V vs. RHE only. This consistent behavior across different redox species suggested the presence of Fermi-level pinning (FLP) located roughly 0.35 V negative to the flat band potential (EFB), as inferred from the photoelectrochemical measurements. Time-resolved microwave conductivity (TRMC) measurements suggested that the bulk material has favorable transport properties, with a long lifetime for photogenerated carriers (about 200 ns) and an associated diffusion length of about 300 nm, suggesting that a photovoltage of 0.8 V can be achieved. Thus, the initially reported band diagram of CuFeO_2_ was refined by EIS (Figure 4c), Mott–Schottky (MS) analysis (Figure 4d) and Kelvin Probe Force Microscopy (KPFM) and X-Ray Photoelectron Spectroscopy (XPS) (Figure 4e), which allowed a comprehensive picture of the band diagram of CuFeO_2_ to be derived (Figure 4f), highlighting the role of a high density of surface states (~10^14^ cm^−2^) at +0.7 V vs. RHE and Fermi-level pinning (FLP) induced by an approximately 10 nm thick continuous layer of metal hydroxide or oxyhydroxide on the surface of CuFeO_2_ [67].

**Figure 5:**
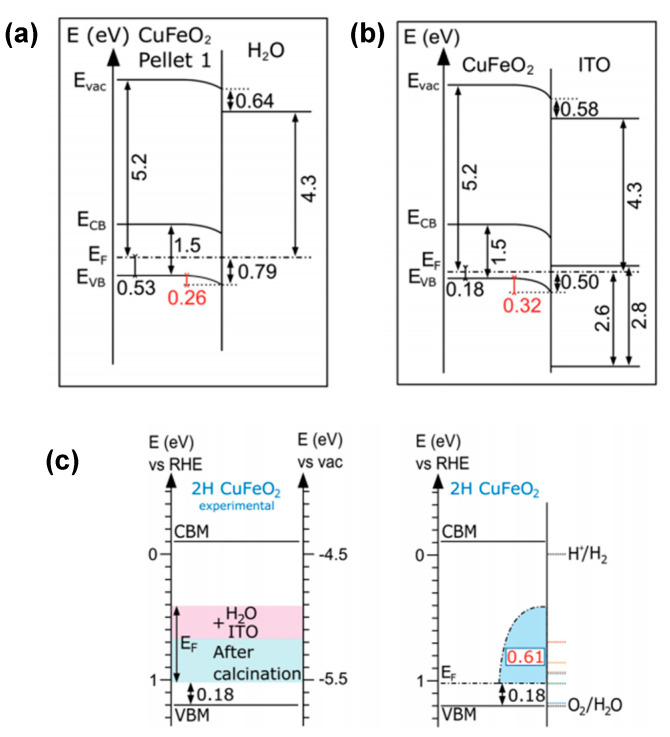
Energy band diagrams of CuFeO_2_−H_2_O (**a**) and the CuFeO_2_−ITO (**b**) interface, revealing the Fermi level tunability in 2H CuFeO_2_ hexagonal nanoplatelet−shaped powder materials. (**c**) Corresponding summary of the experimental Fermi level ranges after interface formation (ITO or water). Reprinted with permission from ref. [69], Copyright (2020), *Advanced Functional Materials*.

In contrast, Hermans et al. [69] proposed that Fermi-level pinning at the Fe^3+^/Fe^2+^ electron polaron formation level intrinsically limits CuFeO_2_ from achieving a sufficient photovoltage. They argued that this is evidenced by the electronic and chemical properties of the CuFeO_2_–ITO and CuFeO_2_–H_2_O interfaces mapped by photoelectron spectroscopy (Figure 5). They concluded that Fermi level positions span across a wide range of 0.61 eV (see Figure 5c). This high degree of Fermi level tunability in CuFeO_2_ has a direct impact on its water reduction efficiency (see Figure 5a,b). The authors hypothesized that the observed Fermi-level pinning was not attributable to surface states but rather resulted from the formation and occupancy of the bulk Fe^3+^/Fe^2+^ polaron level and supported their assumption by similarities in octahedral Fe coordination in Fe_2_O_3_ [89] and CuFeO_2_, a consistent Fermi-level pinning position for various polytypes of CuFeO_2_ [67,71], and observations of similar charge transition levels in BiFeO_3_ at 0.7 eV vs. RHE [89,90]. Thus, the authors considered pinning at the Fe^3+^/Fe^2+^ charge transition level to be a prevalent phenomenon in iron-containing oxides. This can be considered an inherent challenge for CuFeO_2_, which is yet to be addressed by a suitable engineering solution.

## 3. Implemented Strategies for Improving Photoelectrochemical Performance of CuFeO_2_

Follow-up research aimed at addressing the limitations of CuFeO_2_ by engineering solutions to enhance the hydrogen evolution efficiencies of CuFeO_2_ photocathodes by (i) defect engineering, (ii) strategies to enhance light harvesting, or (iii) by heterojunction formation to improve charge separation. The overarching objective of defect engineering is to modulate the concentration of the (majority) charge carriers and to enhance conductivity through extrinsic or intrinsic doping. Secondly, enhancing light harvesting focuses on overcoming challenges such as limited visible light harvesting, low incident photon-to-current conversion efficiency (IPCE), and relatively weak absorption. Lastly, heterojunction formation aims at improving charge carrier separation while diminishing surface recombination. This approach is designed to address issues related to poor charge carrier transport, inadequate charge separation and transport, and the mitigation of unfavorable surface states. In the following sections, these strategies will be examined in detail, exploring their contribution to the enhancement of the efficiency of CuFeO_2_-based photocathodes in PEC water splitting applications.

### 3.1. Defect Engineering

**Figure 6:**
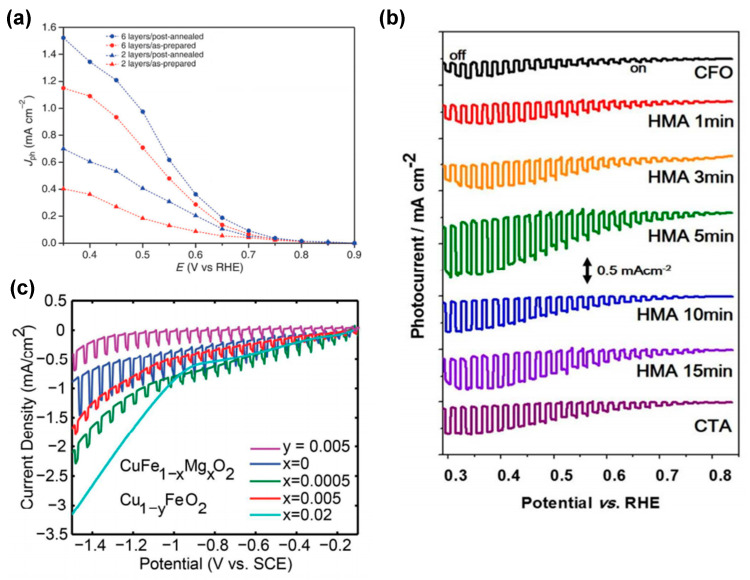
(**a**) Photocurrent (total current minus dark current), J_ph_, as a function of applied potential for a six-layer CuFeO_2_ electrodes obtained in oxygen-saturated 1 M NaOH under one sun illumination before (red) and after (blue) post−annealing at 300 C in air. Reprinted with permission from ref. [71], Copyright (2015), *ChemSusChem*. (**b**) Current (J) -potential (V) curves of untreated CuFeO_2_, and post-treated CuFeO_2_ by CTA and HMA. Reprinted with permission from ref. [80], Copyright (2016), *Chemistry of Materials*. (**c**) Linear sweep voltammetry measurements of CuFe_1-x_Mg_x_O_2_ and Cu_1-y_FeO_2_. Reprinted with permission from ref. [79], Copyright (2017), *Materials Chemistry*.

The influence of oxygen intercalation by air treatment at 300 °C for 1 h on the photoelectrochemical properties of CuFeO_2_ thin films was first investigated by Prévot et al. [28]. The treatment was shown to result in a noticeable improvement in the photocurrents generated, with the best-performing electrode exhibiting a substantial increase in photocurrents, reaching 1.51 mA cm^−2^ at +0.35 V vs. RHE under front illumination and in oxygen-purged 1 M NaOH (Figure 6a). The enhancement was assigned to an increase in the acceptor density of the six-layer electrode after oxygen intercalation by approx. 20%. Annealing strategies were also investigated by Jang et al. [8]. In addition to conventional thermal annealing (CTA), hybrid microwave annealing (HMA) was shown to result in superior oxygen intercalation and partial oxidation of Cu^+^ to Cu^2+^. This process led to a more uniform distribution of Cu^2+^ while simultaneously inducing electron deficiency in Fe (Fe^3+δ^). The HMA-treated electrode exhibited the highest performance, achieving a cathodic photocurrent of −1.3 mA cm^−2^ at 0.4 V_RHE_, which is more than four times the performance of the unannealed CuFeO_2_ (Figure 6b). Furthermore, Mott–Schottky analysis (dark) revealed that the HMA-treated CuFeO_2_ electrode had the highest charge carrier density, which led to enhanced charge separation and carrier density, as also confirmed by XPS and XANES.

In addition to oxygen intercalation, cation doping and fabrication of Cu-deficient CuFeO_2_ electrodes are other common methods employed to increase the carrier concentration and conductivity of Cu-based photoelectrodes [93]. For instance, Wuttig et al. [79] sought to modulate the carrier concentration and thus the photoresponse of CuFeO_2_ photoelectrodes by Mg doping and Cu deficiency, as revealed by Hall effect measurements on pure, Mg-incorporated, and Cu-deficient pellets. The study revealed that pure CuFeO_2_ exhibited the lowest p-type carrier concentration and the highest carrier mobility, resulting in the largest photoresponse with a photocurrent of 0.43 mA/cm^2^ obtained under chopped light illumination (Figure 6c). The conductivity of CuFeO_2_ appeared to be limited by the delafossite defect chemistry, which changes the majority carrier type from p-type to n-type near the Mg solubility limit and at high Cu defect concentrations. The prevalence of n-type defects hinders the ability to achieve high p-type carrier densities, thereby emphasizing the delicate equilibrium between dopant concentrations, defect interactions, and material structure that governs the performance of CuFeO_2_ in PEC applications. In contrast, Jiang and collaborators [81] achieved a notable breakthrough by introducing more acceptor-type states, effectively increasing the concentration of p-type carriers using hydrothermal doping of Mg into CuFeO_2_. Their approach also bolstered the conductivity of CuFeO_2_, facilitating smoother carrier transport for photocathodes based on hexagonal CuFeO_2_ platelets. This advancement was accomplished through a straightforward method, hydrothermal doping of Mg into CuFeO_2_, yet with the optimal composition of 0.1% Mg, a CuFeO_2_ photocurrent density of only 100 μA cm^−2^ was obtained, and higher Mg-dopant concentrations led to a decline in photocurrent density.

### 3.2. Enhancing Light Harvesting

**Figure 7:**
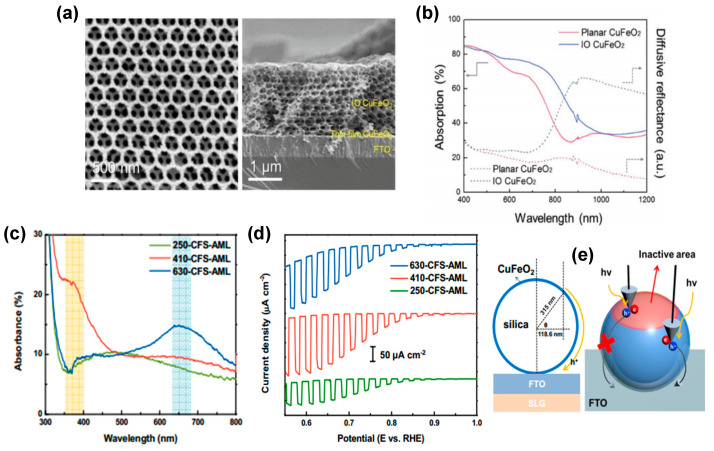
(**a**) Top−view and cross−sectional SEM images of the IO−CuFeO_2_. (**b**) Absorption and diffusive reflectance of the planar and IO−CuFeO_2_ materials. Reprinted with permission from ref. [77], Copyright (2019), *Advanced Functional Materials*. (**c**) Absorbance spectra of a CFS−AML photocathodes. (**d**) LSV of CFS−AML photocathode. Reprinted with permission from ref. [84], Copyright (2017), *ACS Applied Materials & Interfaces*. (**e**) Schematic image of the maximum travel length of the majority carriers.

Overcoming the limitations of CuFeO_2_ delafossite photocathodes has also been addressed using strategies aimed at enhancing light absorption, reducing surface reflection by multiple scattering, and facilitating the formation of samples with a short carrier diffusion length. For example, an inverse opal (IO) nanostructure was proposed by Oh et al. [77] using an opal template and sol infiltration. In comparison with planar CuFeO_2_ photocathodes, the dimensionally ordered IO structure with a hexagonal skeleton and eight layers of a microporous structure (see Figure 7a) exhibits an evident absorption enhancement at 600–800 nm compared to planar CuFeO_2_ (see Figure 7b). IO-CuFeO_2_ photocathodes exhibited nearly twice the PEC performance of the planar CuFeO_2_, with a photocurrent density of 1.05 mA cm^−2^ at 0 V versus RHE.

Oh et al. [84] also developed transparent CuFeO_2_ photoelectrodes showing comparable photocurrent with thin film-based photoelectrodes using an innovative architecture composed of two-dimensional silica microsphere photonic crystals (2D PC) decorated with a thin CuFeO_2_ layer. The concept facilitates the establishment of a diverse range of PC systems, each exhibiting a distinct absorption range. These absorption ranges are attributed to the periodic arrangement of distinct refractive index materials, with the photonic stop band (PSB) of each system contingent on the diameter of the microspheres (see Figure 7c). As pointed out by Oh et al., 2D PC systems containing microspheres@CuFeO_2_ with a diameter of 550 nm would likely facilitate the generation of higher photocurrents. This is due to the more favorable location of their PSB, which would allow for better utilization of incoming photons and collection of photogenerated carriers. In conclusion, the research conducted by Oh et al. indicates that CuFeO_2_-decorated microsphere photocathodes, characterized by their visible light transmittance (76.4%), maintain the capacity for photo-current generation (0.2 mA cm⁻^2^ at 0.6 V vs. RHE, refer to Figure 7d). This property enables their integration with small band gap semiconductors within a tandem device configuration [94].

### 3.3. Improving Charge Carrier Separation by Heterojunction Formation

**Figure 8:**
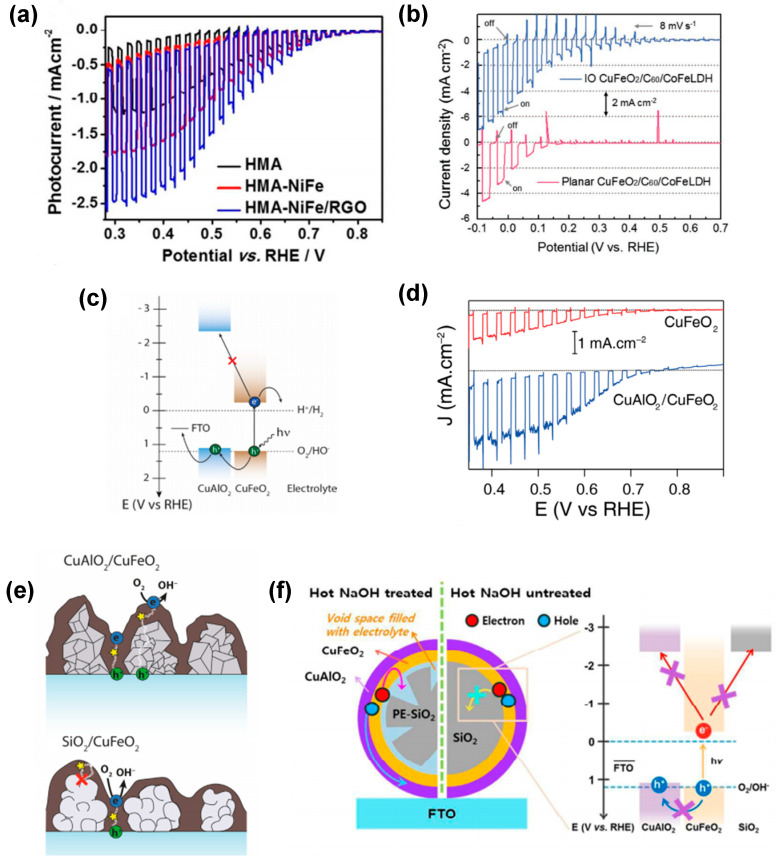
(**a**) J−V curves of HMA, HMA−NiFe, and HMA−NiFe/RGO. Reprinted with permission from ref. [80], Copyright (2016), *Chemistry of Materials*. (**b**) Linear sweep voltammograms of the IO and planar CuFeO_2_ photocathodes after modification by the electrocatalyst. Reprinted with permission from ref. [77], Copyright (2019), *Advanced Functional Materials*. (**c**) Simplified energy diagram of the host−guest CuAlO_2_/CuFeO_2_ electrode. (**d**) Comparison of J−V curves obtained for optimized CuAlO_2_/CuFeO_2_ (blue trace) and CuFeO_2_ (red trace) electrodes. Reprinted with permission from ref. [68], Copyright (2016), *Materials Chemistry*. (**e**) Scheme of the possible pathways that photogenerated charges can take inside CuAlO_2_/CuFeO_2_ and SiO_2_/CuFeO_2_ electrodes. Reprinted with permission from ref. [83], Copyright (2018), *Nanoscale*. (**f**) Schematic illustration of the electron transfer pathways at the CuFeO_2_−electrolyte interface for 2D opal-based photocathodes.

The formation of heterojunctions to increase the charge separation of photogenerated charge carriers is often proposed in combination with other strategies. For example, Jang et al. [80] used HMA-treated CuFeO_2_ electrodes for coupling with a hydrogen evolution reaction (HER) electrocatalyst onto its surface. The authors particularly used nickel-iron layered double hydroxide (NiFe LDH) prepared by a one-step hydrothermal method and deposited on HMA-CuFeO_2_ photocathodes (HMA-NiFe). Additionally, a combination of NiFe LDH and reduced graphene oxide (HMA-NiFe/rGO) was investigated. Both modifications (Figure 8a) led to drastic improvements in photocurrent generation up to −2.4 mA cm^−2^ at 0.4 VRHE, being a 7–8 fold and 1.5–2 fold increase for HMA-NiFe/rGO relative to unannealed CuFeO_2_ and HMA-CuFeO_2_ photocathodes, respectively. Oh et al. [77] modified IO-CuFeO_2_ and planar CuFeO_2_ photocathodes with a CoFe LDH catalyst and a C_60_ layer, respectively. The determined photocurrent density of −4.86 mA cm^−2^ at 0 V vs. RHE (Figure 8b) was shown to be a factor of 2 larger than the HER performance of the planar CuFeO_2_/C_60_/CoFe LDH photocathode. IPCE measurements revealed that the IO-CuFeO_2_-based photocathode exhibited a high IPCE value of 17.5% at 600 nm due to both increased light absorption and enhanced charge transfer/extraction.

As highlighted above, the high density of surface states within nano-sized CuFeO_2_ is assumed to enhance recombination of photoexcited electrons. To address this problem, Aqaei et al. [82] introduced a diamond-like carbon (DLC) scaffold with plasmonic gold (Au) nanoparticles (NPs) atop CuFeO_2_. These Au@DLC/CuFeO_2_ samples were fabricated using RF-sputtering and RF-Plasma Enhanced Chemical Vapor Deposition (PECVD); however, only a minor improvement in the PEC performance of CuFeO_2_ was revealed, which was assigned to the passivation of electron traps.

The challenge of reconciling minority carrier transport and light absorption has been a central concern in enhancing the performance of various photoelectrodes, including hematite photoanodes. Host-guest approaches, in which an ultra-thin layer of the light absorber is deposited onto a high surface area scaffold with suitable electronic properties, have been shown to address this issue effectively. This approach is well-known for its ability to facilitate the decoupling of light absorption and charge transfer. This, in turn, has been shown to mitigate charge carrier recombination, thus leading to enhanced photocurrents [95,96]. Applying this approach to CuFeO_2_ requires identification of a p-type material fulfilling four fundamental criteria: possessing (1) a high transparency as observed for wide band gap (large E_g_) materials; (2) substantial p-type (hole) conductivity; (3) valence and conduction band energy levels exceeding those of CuFeO_2_ to impede back electron transfer and facilitate the efficient transfer of holes from the absorber to the scaffold; (4) high stability under the conditions required for CuFeO_2_ preparation and for PEC testing. Prévot et al. [68] and Oh et al. [83] successfully applied the host-guest composite electrode strategy to CuFeO_2_ photoanodes using a p-type CuAlO_2_ scaffold. Indeed, the optoelectronic properties of CuAlO_2_ (Figure 8c) have been demonstrated to be advantageous, in accordance with its visible light transparency (Eg = 3.5 eV) and a flat band potential that is 0.1 eV higher than that of CuFeO_2_. Given this offset, a selective extraction of photogenerated holes towards the substrate at the CuFeO_2_–CuAlO_2_ interface is predicted. The thickness of the host and guest layers was independently varied, thus enabling the successful fabrication of a host-guest electrode on a 2 µm CuAlO_2_ scaffold that contains approximately 2.2 times more CuFeO_2_ than the most efficient bare CuFeO_2_ electrode (prepared directly on FTO) [68]. Using the scaffold layer with a thickness of 2 µm resulted in a 2.4-fold increase in photocurrent density in the presence of O_2_ as a sacrificial electron scavenger compared to bare CuFeO_2_, i.e., a photocurrent density of 2.4 mA cm^−2^ at +0.4 V vs. RHE (Figure 8d) was obtained. A comparison of the host-guest strategy with CuFeO_2_ layers prepared on an insulating SiO_2_ scaffold further suggested that CuAlO_2_ facilitated improved charge transport rather than merely decreasing recombination rates when compared to the CuFeO_2_ film alone (Figure 8e). In addition, the host-guest approach was also combined with the transparent 2D opal photocathode system [84]. By introducing a double-shelled heterojunction configuration with inner CuFeO_2_ and outer CuAlO_2_ shells on silica microsphere scaffolds [83], efficient charge transfer (Figure 8f) and a 9-fold enhancement in photoresponse in comparison to single-shelled counterparts were achieved. In this design, issues related to the blockage of electrolyte were mitigated by the design of a photoelectrode based on partially etched silica microspheres (PE-SiO_2_).

## 4. Fermi-Level Pinning in CuFeO_2_: Lessons from Other Photoelectrodes

The upper limits of the Fermi energy in CuFeO_2_ have been determined to be approximately 0.8 eV [69], thus indicating a relatively constrained range for a material that possesses a 1.5 eV band gap (Figure 3b). This limitation impacts the photovoltage of a photocathode, as it is dictated by the splitting of the quasi-Fermi levels at the electron–hole contacts. To fully harness the band gap potential of CuFeO_2_ for water splitting, it is necessary to develop a more profound understanding of Fermi-level pinning phenomena in CuFeO_2_ and other materials, as well as strategies to mitigate it. This section focuses on elucidating both intrinsic contributions and other mechanisms of Fermi-level pinning in materials like CuFeO_2_ and their implications for optimizing performance. To clarify, intrinsic contributions to Fermi-level pinning are inherent to the material itself. These include phenomena such as Metal-Induced Gap States (MIGS) and electrochemical changes within the semiconductor, which arise from its fundamental structure and bonding. In contrast, other mechanisms involve external factors, such as crystallographic defects or foreign atoms introduced during material processing, which can alter the electronic properties. Differentiating between these contributions is key to developing targeted strategies for mitigating Fermi-level pinning and improving the PEC performance of CuFeO_2_.

### 4.1. Intrinsic Contributions to Fermi-Level Pinning

Interface states affecting the barrier height can emerge even at defect-free surfaces when interfaces between semiconductors and metals, as well as at interfaces between two semiconductors, are established. A metal in contact with the surface of a semiconductor results in an overlap of the wave function of valence electrons of the semiconductor and the metal, thereby aligning their Fermi levels. This alignment gives rise to gap states known as Metal-Induced Gap States (MIGS), which penetrate deeper into the semiconductor and are responsible for pinning the surface energy state, irrespective of the type of metal used [97,98]. These MIGS play a crucial role in Fermi-level pinning (FLP) and facilitate electron and hole tunneling into the semiconductor [99]. The barriers for tunneling at semiconductor–metal interfaces are determined by the Schottky barriers for electrons and holes, while for semiconductor heterojunctions, they are defined by the valence and conduction band offsets, respectively. The nature of interface states is dependent on the character of the chemical bond developed between constituent atoms; ionic materials display little or no Fermi-level stabilization at the interface, while covalent materials display virtually complete stabilization [100,101]. This phenomenon suggests a dependence on the electronegativity of the metal, reflecting the local charge transfer related to the chemical bonds at the semiconductor–metal interface, rather than relying on specific surface properties of the metal. Therefore, the degree of Fermi-level pinning is often characterized in terms of an index of interface behavior S, defined as follows:

(3) $$\phi_{B}=S\times \chi_{m},$$

where $\phi_{B}$ represents Schottky barrier heights and $\chi_{m}$ is the electronegativity of the metal forming the barrier.

The index of interface behavior S is small for strong Fermi-level pinning, a characteristic notably pronounced in semiconductors with strong covalent bonding character [102]. This is evident in elemental semiconductors such as carbon (C), silicon (Si), germanium (Ge), and group III–V compounds like gallium arsenide (GaAs) and others, where S is approximately 0.1. Conversely, semiconductors with more ionic bonds typically exhibit weaker Fermi-level pinning, with S around 1.

In addition to Metal-Induced Gap States (MIGS), electrochemical reduction or oxidation of a material is another intrinsic contributor responsible for FLP [103]. These valence changes of cationic or anionic species effectively constrain the Fermi energy within a material, as the respective concentration of the defects is equal to that of the atoms. Recently, Suzuki et al. [104] demonstrated limited Fermi shifts in β-CuGaO_2_ to ∼0.8 eV, significantly smaller than its band gap of 1.5 eV. The origin of the Fermi-level pinning, similar to that observed at Cu_2_O–ZnO interfaces [105], is related to the electrochemical oxidation/reduction of Cu. Observations of upper limits of the Fermi energy have also been made in Fe_2_O_3_ [89], BiFeO_3_ [90], and BiVO_4_ [106], where direct spectroscopic evidence for the reduction of Fe or V was obtained. In the case of CuFeO_2_, the electrochemical reduction of Fe, which corresponds to a valence change from Fe^3+^ to Fe^2+^, is anticipated to constitute a fundamental constraint on the Fermi level energy.

### 4.2. Other Mechanisms Contributing to Fermi-Level Pinning

**Figure 9:**
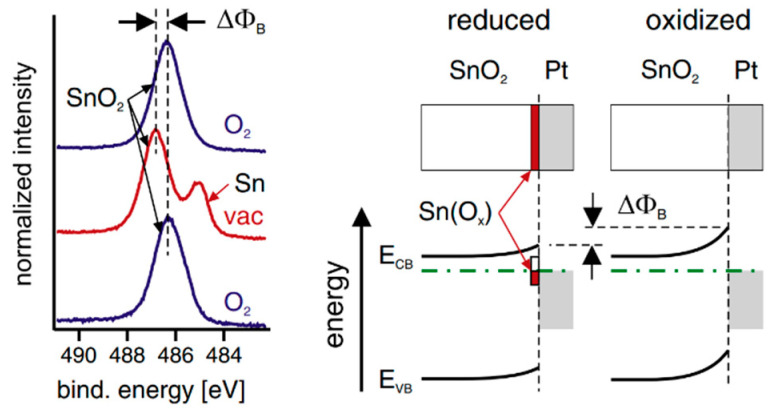
(**Left**) Sn 3d photoelectron spectra recorded from a polycrystalline SnO_2_ thin film coated with ~2 nm Pt. (**Right**) Energy band diagrams and schematic interface chemical structures of oxidized and reduced SnO_2_–Pt interfaces. Reprinted with permission from ref. [115], Copyright (2012), *Thin Solid Films*.

Crystallographic defects can also induce Fermi-level pinning, with point defects being a typical example that induce Fermi-level pinning in ionic compounds. For instance, oxygen vacancies in ZnO form deep donor states [107,108], while copper vacancies in CuGaSe_2_ contribute to self-compensation defects [109]. Moreover, extrinsic states such as crystallographic defects (point defects and dislocations) can impact barrier heights at semiconductor–metal interfaces [110]. This influence can arise from factors like lattice mismatch [111] or chemical reactions during interface formation. For instance, the oxygen content at the semiconductor–metal interface can vary significantly [112], and the deposition of metals onto semiconductor surfaces can lead to defect formation due to the release of heat during condensation. Rao et al. [113] calculated the heat of condensation of Pt onto BaTiO_3_ to be approximately 4 eV, a value exceeding the energy required to create oxygen vacancies in most conducting oxides. This contrasts with the energy required to create oxygen vacancies in conducting oxides, which varies depending on the specific material. For example, in BiFeO_3_, the oxygen vacancy energy level is calculated to be 0.6 eV below the conduction band edge [114]. These defects are likely to be controlled through careful selection of contact materials and processing techniques during interface fabrication. Their concentration can be adjusted through post-deposition treatments, such as annealing oxide–metal interfaces in oxidizing atmospheres which increases the barrier height for electrons, whereas reducing atmospheres result in a lower barrier. These changes in barrier height, which can be substantial (up to 1 eV), may occur even at room temperature, particularly with catalytically active contact metals like Pt. For example, photoelectron spectra of a SnO_2_ surface coated with a ~2 nm Pt film show a fully oxidized surface when exposed to an oxygen atmosphere, while metallic Sn species occur under vacuum. This reversible switching between oxidized and reduced states demonstrates the potential for controlling interface properties through atmospheric manipulation, as shown in Figure 9 [115].

## 5. Suggested Strategies for Enhancing CuFeO_2_ PEC Performance

In Section 3, we provided a comprehensive review of the strategies that have been implemented to enhance CuFeO_2_ photocathodes. These strategies encompass defect engineering, improving light harvesting, and utilizing heterojunctions. However, as discussed in Section 4, the primary challenge for CuFeO_2_ photocathodes is Fermi-level pinning (FLP), which limits the material’s performance in photoelectrochemical (PEC) applications. To address this challenge, it is essential to explore strategies that specifically target FLP mitigation. In this section, we present three potential strategies to enhance the PEC performance of CuFeO_2_.

**Figure 10:**
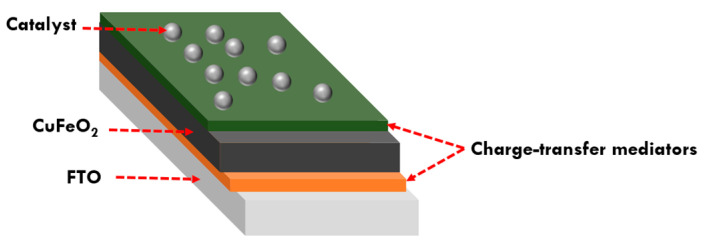
Schematic representation of an integrated CuFeO_2_-based photocathode design.

Recent research focusing on the integration of mediators such as conductive polymers, metal nanoparticles, or molecular catalysts into selective contacts for photocathodes represents a notable advancement in the field [116]. These mediators can enhance charge transfer, facilitate electron or hole transport, and improve overall efficiency in PEC applications. While previous studies have examined hole or electron selective contacts individually, integration of both types of contact has thus far not been reported. Notably, the integration of a hole-selective contact between FTO and CuFeO_2_, which is essential for impeding back electron transfer and facilitating hole transfer to FTO, is a significant development. Additionally, the integration of an electron-selective contact between CuFeO_2_ and the catalyst or electrolyte, aimed at enhancing charge transfer and overcoming the hindrance posed by the high density of surface states within CuFeO_2_, is noteworthy. These integrated systems, driven by electrochemical potential gradients and employing multimediators, hold immense promise for significantly enhancing charge separation and transportation (Figure 10).

Despite the fact that CuFeO_2_ has already been shown to exhibit favorable charge transport properties (see Table 3) in comparison to other photoelectrodes, electrochemical impedance spectroscopy (EIS) measurements further support the substantial impact of selective contacts [117]. It is therefore concluded that such advancements have the potential to lead to improved overall performance in the performance of Cu delafossite photoelectrodes.

Addressing the substrate’s role as a current collector is another critical factor, particularly given that the majority of reported CuFeO_2_ photocathodes deposit CuFeO_2_ directly onto FTO substrates. However, existing literature indicates the inactivity of iron-containing metal oxide films during operation caused by direct interaction and interface formation with fluorine-doped tin oxide (FTO) [118,119]. For instance, in hematite films like α-Fe_2_O_3_, this interaction widens Fe 3d−O 2p hybridized states. This has been shown to result in decreased p-d orbital hybridization and increased recombination of photogenerated charge carriers [120,121,122]. Furthermore, photoelectrochemical losses have been linked to the low degree of crystallinity of deposited iron-containing metal oxides on FTO, which correlates with the degree of light absorption and photoelectrochemical water oxidation performance [123,124]. These findings suggest that interfacial layers between the absorber and current collector significantly impact electrode assembly transport properties. To overcome these drawbacks, exploring alternative current collectors such as copper (Cu), graphene, or conductive polymers represents a promising avenue for enhancing photoelectrode performance. For instance, copper has been utilized due to its excellent electrical conductivity and compatibility with various semiconductor materials [125]. Research by Fu et al. demonstrated that using graphene as a current collector improved charge transport and reduced recombination losses in CuFeO_2_-based photocathodes [86]. Additionally, the integration of a buffer layer, such as titanium dioxide (TiO_2_) or zinc oxide (ZnO), between the FTO substrate and the iron-containing metal oxide can significantly enhance performance. Recent studies have demonstrated that incorporating a TiO_2_ buffer layer improved the crystallinity and light absorption of α-Fe_2_O_3_ films, leading to enhanced photocatalytic activity [126].

If the hypothesis that the constraint Fermi level energy is caused by electro-chemical reduction of Fe is correct, then the full utilization of CuFeO_2_′s band gap as a photocathode may prove challenging. In such a scenario, it becomes necessary to explore alternative mechanisms for charge separation, moving away from conventional approaches such as p-n junctions or heterojunctions, which are limited by the electronic bandgap of the semiconductor. One promising avenue involves the exploitation of the ferroelectric property of CuFeO_2_. In this context, Yang et al. [127] have demonstrated the role of ferroelectric domain walls in enhancing charge separation and generating a larger photovoltage in BiFeO_3_ thin films. Their findings revealed significant differences in photovoltaic behavior depending on the orientation of domain walls relative to the electrodes, resulting in notably higher photovoltages than the bandgap alone would suggest. They emphasize the importance of controlling the domain structure in BiFeO_3_ films by growing them on annealed and un-annealed DyScO_3_ (DSO) substrates. Additionally, they demonstrate that the photovoltaic effect can be controlled by applying electric fields to induce ferroelectric domain switching, creating a system with perpendicular domain walls and enhancing the photovoltaic effect.

## 6. Concluding Remarks

In the context of photoelectrochemical water splitting, Cu-based metal oxide photocathodes, notably CuFeO_2_, have emerged as a prominent class of materials due to their advantageous properties, including narrow bandgaps, cost-effectiveness, and favorable band-edge positions that are conducive to water splitting. CuFeO_2_ is notable for its responsiveness to visible light, exhibiting multi-band absorption and an anticipated high photovoltage owing to its theoretical Fermi level positioned at 1.5 V vs. RHE. However, despite these promising properties, unprotected CuFeO_2_ photocathodes face significant challenges, including exceptionally low photocurrent and difficulties in effectively driving hydrogen production during water reduction. A primary challenge in addressing this issue is the difficulty in synthesizing high-quality, pure-phase CuFeO_2_ on FTO substrates, which can be attributed to the inherent complexities of the Cu–Fe–O system, leading to the formation of undesirable phases. Furthermore, the presence of bulk or interface defects, situated approximately 0.35 eV above the flat band potential, leads to Fermi-level pinning at the semiconductor–liquid junction. This pinning significantly restricts the photovoltage of bare CuFeO_2_ to 0.35 V. This limitation poses a major obstacle to the advancement of unbiased tandem devices incorporating CuFeO_2_ photocathodes, necessitating substantial enhancements in CuFeO_2_′s photovoltage. This review comprehensively explores recent advancements in earth-abundant CuFeO_2_ photocathode design, offering insights into the current understanding of key factors diminishing the STH efficiencies, such as Fermi-level pinning, and pointing out potential strategies to overcome these challenges, among which we consider careful design of hole and electron mediator layers as being most promising, all with the aim of advancing the potential of CuFeO_2_ as a practical material for PEC water splitting for solar hydrogen production.

## Figures and Tables

**Table 1:** Comparison of photocathode materials for PEC water splitting. Note that the table only provides reference to unprotected photocathodes.

Material	Bandgap (eV)	Photocurrent Density (mA cm^−2^)	Stability (Hours)	Solar-to-Hydrogen Conversion Efficiency [%]	Toxicity	References
CuFeO_2_	1.55	−1.51 at +0.4 V_RHE_	50	10 at 400 nm	Low	[71]
p-Si	1.1–1.3	−0.25 at +0 V_RHE_		0.03	Low	[72]
CdTe	1.5	−0.01 at 0.1 V_RHE_			High	[73]
Cu(In,Ga)S_2_	1.0–1.7	−5.25 at −0.4 V_RHE_		5.4	Moderate	[74]
CuBi_2_O_4_	1.8	−0.6 at 0.3 V_RHE_	8		Low	[75]

**Table 2:** Comparison of the PEC water reduction performances of reported CuFeO_2_-based photocathodes.

Reference	Device Structure	Photocurrent Density (mA cm^−2^)	Onset Potential (V_RHE_)	Stability	IPCE (%)	Faradaic Efficiency (%)	Electrolyte, Light Source
Wuttig et al. [79]	CuFeO_2_	−0.43 at −1.4 V_SCE_	−0.2 V vs. SCE	8 h (minimal self-reduction)			0.1 M NaHCO_3_, 1 sun (100 mWcm^−2^)
Jang et al. [80]	CuFeO_2_	−0.3 at 0.4 V_RHE_	0.65		3 at 300 nm		1 M NaOH–Ar, 1 sun (100 mWcm^−2^)
	CuFeO_2_-CTA	−0.62 at 0.4 V_RHE_	0.65		7 at 300 nm		1 M NaOH–Ar, 1 sun (100 mWcm^−2^)
	CuFeO_2_-HMA	−1.3 at 0.4 V_RHE_	0.65		14 at 300 nm		1 M NaOH–Ar, 1 sun (100 mWcm^−2^)
	CuFeO_2_-HMA-NiFe	−1.7 at 0.4 V_RHE_	0.65				1 M NaOH–Ar, 1 sun (100 mWcm^−2^)
	CuFeO_2_-HMA-NiFe/RGO	−2.4 at 0.4 V_RHE_	0.65		22 at 300 nm	94	1 M NaOH–Ar, 1 sun (100 mW cm^−2^)
Jiang et al. [81]	CuFeO_2_	−0.1 at 0.4 V_SCE_					1 M NaOH, 1 sun (100 mW cm^−2^)
Prévot et al. [71]	CuFeO_2_	−0.025 at +0.4 V_RHE_	0.9				Ar-purged 1 M NaOH, 1 sun (100 mWcm^−2^)
	CuFeO_2_	−1.51 at +0.4 V_RHE_	0.9	40 h (stability with no reduction)	10 at 400 nm		1 M NaOH purged with O_2_, 1 sun (100 mWcm^−2^)
	CuFeO_2_	−0.5 at +0.4 V_RHE_	0.9				MV^2+^ (0.01 M in 1 M NaOH), 1 sun (100 mWcm^−2^)
	CuFeO_2_/AZO/TiO_2_/Pt	−0.8 at −0.2 V_RHE_	0.4	50 min (stability with no reduction)			Ag-purged pH 6.1 (0.5 m Na_2_SO_4_), 1 sun (100 mWcm^−2^)
Aqaei et al. [82]	CuFeO_2_	−0.6 at 0.4 V_RHE_	0.9				0.5 M KOH, Xe light source with 400 mW cm^−2^ intensity
	Au@DLC/CuFeO_2_	−0.8 at 0.4 V_RHE_	0.9				0.5 M KOH, Xe light source with 400 mW cm^−2^ intensity
Oh et al. [83]	SiO_2_@CuFeO_2_	−0.3 at 0.6 V_RHE_	0.9				1 M NaOH, 1 sun (100 mW cm^2^)
	PE-SiO_2_@CuFeO_2_	−0.12 at 0.6 V_RHE_	0.9		2 at 400 nm		1 M NaOH, 1 sun (100 mWcm^−2^)
	SiO_2_@CuFeO_2_@CuAlO_2_	−0.19 at 0.6 V_RHE_	0.9				1 M NaOH, 1 sun (100 mWcm^−2^)
	PE-SiO_2_@CuFeO_2_@CuAlO_2_	−1.09 at 0.6 V_RHE_	0.9		10 at 400 nm		1 M NaOH, 1 sun (100 mWcm^−2^)
Oh et al. [77]	CuFeO_2_	−0.51 at 0 V_RHE_	0.12				Ar-purged 1 M NaOH (pH 13.5), 1 sun (100 mWcm^−2^)
	CuFeO_2_/C_60_/ CoFe LDH	−2.11 at 0 V_RHE_	0.15		25 at 400 nm 4.8 at 600 nm		Ar-purged 1 M NaOH (pH 13.5), 1 sun (100 mWcm^−2^)
	IO CuFeO_2_	−1.05 at 0 V_RHE_	0.65				Ar-purged 1 M NaOH (pH 13.5), 1 sun (100 mWcm^−2^)
	IO CuFeO_2_/C_60_/ CoFe LDH	−5.2 at −0.1 V_RHE_	0.65		17.5 at 600 nm		Ar-purged 1 M NaOH (pH 13.5), 1 sun (100 mWcm^−2^)
Oh et al. [84]	Microsphere@CuFeO_2_	−0.07 at 0.6 V_RHE_	0.95		4.5 at 400 nm		Ar-purged 1 M NaOH (pH 14), 1 sun (100 mWcm^−2^)
	Microsphere@CuFeO_2_	−0.2 at 0.6 V_RHE_	0.9		4.5 at 400 nm		1 M NaOH purged with O_2_, 1 sun (100 mW cm^−2^)
Prévot et al. [68]	CuFeO_2_	−1 at 0.35 V_RHE_	0.75		10 at 400 nm		1 M NaOH purged with O_2_, 1 sun (100 mW cm^−2^)
	CuAlO_2_/ CuFeO_2_	−2.4 at 0.35 V_RHE_	0.75		20 at 400 nm		1 M NaOH purged with O_2_, 1 sun (100 mW cm^−2^)
Bouziani et al. [85]	CuFeO_2_	−0.34 at 0.44 V_RHE_		10 min (slight decrease)			1 M NaOH, 1 sun (100 mW cm^−2^)
Fu et al. [86]	CuFeO_2_	−0.04 at 0.44 V_RHE_		10 min (slight decrease)	1.85 at 350 nm		0.1 M Na_2_SO_4_, 300 W xenon lamp
Präg et al. [87]	CuFeO_2_	0.9 at 0.2 V_RHE_		10 min (slight decrease)	14 at 340 nm		1 M NaOH, 1 sun (100 mW cm^−2^)

**Table 3:** Overview of relevant parameters of the EIS measurements on CuFeO_2_-based photocathodes.

Reference	Device Structure	Series Resistance R_S_ (Ωcm^2^)	Charge- Transfer Resistance R_CT_ (Ωcm^2^)	Constant Phase Element (CPE) C_CT_ (Fcm^−2^)	Electrolyte, Light Source
Oh, Yunjung et al. [83]	PE-SiO_2_@CuFeO_2_	3.55	12992	1.93 × 10^−5^	1 M NaOH, 1 sun
	PE-SiO_2_@CuFeO_2_@CuAlO_2_	1.91	1754	2.65 × 10^−5^	1 M NaOH, 1 sun
Oh, Yunjung et al. [77]	CuFeO_2_/C_60_/CoFe LDH	5.7	370.6	5.5 × 10^−3^	1 M NaOH, 1 sun
	IO CuFeO_2_/C_60_/CoFe LDH	3.4	126.8	1.2 × 10^−2^	1 M NaOH, 1 sun

## Data Availability

No new data were created or analyzed in this study.

## Abbreviations

The following abbreviations are used in this manuscript:

PEC	Photoelectrochemical
**IPCE**	Incident Photon-to-Current Efficiency
**HER**	Hydrogen Evolution Reaction
**OER**	Oxygen Evolution Reaction
**STH**	Solar-to-Hydrogen
**EIS**	Electrochemical Impedance Spectroscopy
**TRMC**	Time-Resolved Microwave Conductivity
**MS**	Mott–Schottky
**KPFM**	Kelvin Probe Force Microscopy
**XPS**	X-Ray Photoelectron Spectroscopy
**HMA**	Hybrid Microwave Annealing
**CTA**	Conventional Thermal Annealing
**LDH**	Layered Double Hydroxide **DLC**
**DLC**	Diamond-Like Carbon
**NPs**	Nanoparticles
**PSB**	Photonic Stop Band
**FLP**	Fermi Level Pinning
**MIGS**	Metal-Induced Gap States
**FTO**	Fluorine-Doped Tin Oxide
**TiO_2_**	Titanium Dioxide
**ZnO**	Zinc Oxide
**DSO**	DyScO_3_
**CFO**	CuFeO_2_
